# Automated analysis of written language in the three variants of primary progressive aphasia

**DOI:** 10.1093/braincomms/fcad202

**Published:** 2023-07-20

**Authors:** Sylvia Josephy-Hernandez, Neguine Rezaii, Amelia Jones, Emmaleigh Loyer, Daisy Hochberg, Megan Quimby, Bonnie Wong, Bradford C Dickerson

**Affiliations:** Frontotemporal Disorders Unit, Department of Neurology, Massachusetts General Hospital & Harvard Medical School, Boston, MA 02114, USA; Frontotemporal Disorders Unit, Department of Neurology, Massachusetts General Hospital & Harvard Medical School, Boston, MA 02114, USA; Frontotemporal Disorders Unit, Department of Neurology, Massachusetts General Hospital & Harvard Medical School, Boston, MA 02114, USA; Speech and Language Pathology Department, Spaulding Rehabilitation Hospital, Charlestown, MA 02129, USA; Frontotemporal Disorders Unit, Department of Neurology, Massachusetts General Hospital & Harvard Medical School, Boston, MA 02114, USA; Frontotemporal Disorders Unit, Department of Neurology, Massachusetts General Hospital & Harvard Medical School, Boston, MA 02114, USA; Frontotemporal Disorders Unit, Department of Neurology, Massachusetts General Hospital & Harvard Medical School, Boston, MA 02114, USA; Frontotemporal Disorders Unit, Department of Neurology, Massachusetts General Hospital & Harvard Medical School, Boston, MA 02114, USA

**Keywords:** writing, PPA, informativeness, content, automated

## Abstract

Despite the important role of written language in everyday life, abnormalities in functional written communication have been sparsely investigated in primary progressive aphasia. Prior studies have analysed written language separately in each of the three variants of primary progressive aphasia—but have rarely compared them to each other or to spoken language. Manual analysis of written language can be a time-consuming process. We therefore developed a program that quantifies content units and total units in written or transcribed language samples. We analysed written and spoken descriptions of the Western Aphasia Battery picnic scene, based on a predefined content unit corpus. We calculated the ratio of content units to units as a measure of content density. Our cohort included 115 participants (20 controls for written, 20 controls for spoken, 28 participants with nonfluent variant primary progressive aphasia, 30 for logopenic variant and 17 for semantic variant). Our program identified content units with a validity of 99.7% (95%CI 99.5–99.8). All patients wrote fewer units than controls (*P* < 0.001). Patients with the logopenic variant (*P* = 0.013) and the semantic variant (0.004) wrote fewer content units than controls. The content unit-to-unit ratio was higher in the nonfluent and semantic variants than controls (*P* = 0.019), but no difference in the logopenic variant (*P* = 0.962). Participants with the logopenic (*P* < 0.001) and semantic (*P* = 0.04) variants produced fewer content units in written compared to spoken descriptions. All variants produced fewer units in written samples compared to spoken (*P* < 0.001). However, due to a relatively smaller decrease in written content units, we observed a larger content unit-to-unit ratio in writing over speech (*P* < 0.001). Written and spoken content units (*r* = 0.5, *P* = 0.009) and total units (*r* = 0.64, *P* < 0.001) were significantly correlated in patients with nonfluent variant, but this was not the case for logopenic or semantic. Considering all patients with primary progressive aphasia, fewer content units were produced in those with greater aphasia severity (Progressive Aphasia Severity Scale Sum of Boxes, *r* = −0.24, *P* = 0.04) and dementia severity (Clinical Dementia Rating scale Sum of Boxes, *r* = −0.34, *P* = 0.004). In conclusion, we observed reduced written content in patients with primary progressive aphasia compared to controls, with a preference for content over non-content units in patients with the nonfluent and semantic variants. We observed a similar ‘telegraphic’ style in both language modalities in patients with the nonfluent variant. Lastly, we show how our program provides a time-efficient tool, which could enable feedback and tracking of writing as an important feature of language and cognition.

## Teaser

Josephy-Hernandez *et al.* report on an efficient automated program to analyse written descriptions by patients with primary progressive aphasia. The analysis reveals differences in how content is conveyed in patients’ writing versus controls or spoken descriptions. Both content and overall output decrease as disease worsens.

## Graphical Abstract

**Graphical Abstract:**
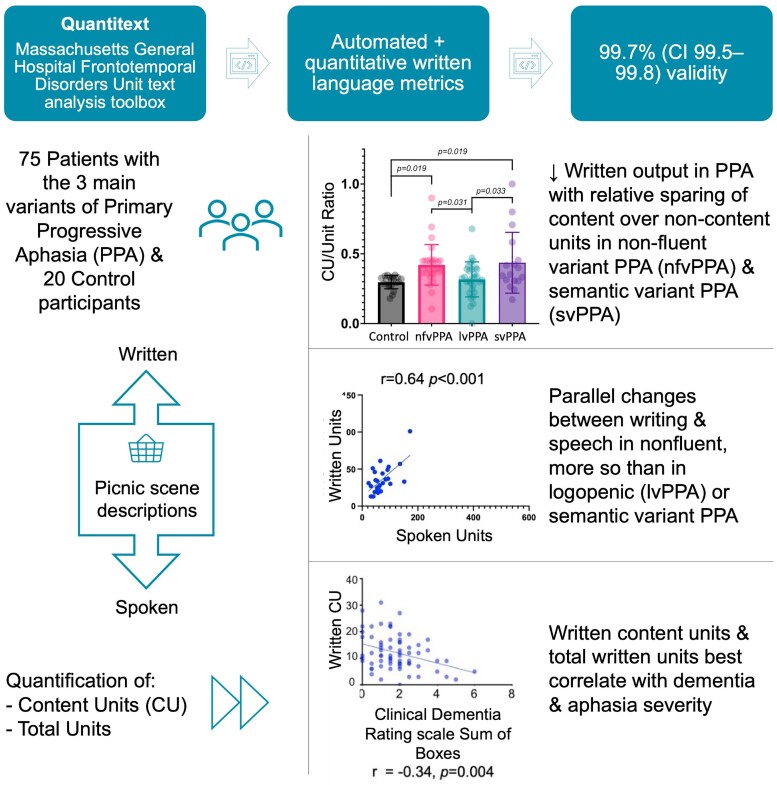

## Introduction

Primary progressive aphasia (PPA) is a clinical syndrome in which aphasia is the initial predominant symptom, usually resulting from Frontotemporal Lobar Degeneration or Alzheimer’s disease (AD).^1^ The characteristics of aphasia in PPA are heterogeneous, with many patients presenting with a profile of language impairments that can be classified into one of three subtypes: the nonfluent/agrammatic variant (nfvPPA), the logopenic variant (lvPPA), or the semantic variant (svPPA).^2^ The characterization and classification of PPA patients’ aphasia are primarily done based on clinical history taken from the patient and an informant, interview of the patient and formal assessment of spoken language. However, in our experience, patients commonly have written language abnormalities as well, and some have predominant alexia^3,4^ or agraphia.^5^ For the three major PPA variants, the only written language characteristic that is part of the diagnostic criteria is surface dyslexia or dysgraphia in svPPA.^2^ Despite the important role of written language in contemporary everyday life (especially in societies dominated by email, online posts and text message communication), abnormalities in functional written communication have been sparsely investigated in PPA. The characterization and measurement of strengths and weaknesses in written communication should be part of the assessment of patients with PPA because some patients may be able to communicate more effectively through some form of writing or typing than through speech. Ultimately, the comparison of spoken and written language output and content in a patient with PPA may offer clinicians opportunities to develop compensatory strategies to maximize functional communication.

Most studies of writing impairments in PPA have focused on spelling, and very few have investigated functional communication. In a comparison of written picture descriptions in patients with nfvPPA to patients with progressive supranuclear palsy (PSP) and controls, patients with nfvPPA showed reduced length, speed and information units compared to controls,^6^ as well as reduced number of written words compared to PSP.^7^ Long-term follow-up of written language in one patient with nfvPPA also showed a decrease in written word output and amount of information, as well as a decrease in sentence complexity over time and increased dependence on nouns over verbs.^8^ A study comparing written picture descriptions of patients with lvPPA to those of amnestic mild cognitive impairment (MCI) and dementia likely due to Alzheimer’s disease reported that patients with lvPPA had increased letter insertion errors and a higher verb use.^9^ A case study of a patient with an unspecified type of PPA and atrophy in the left inferior frontal gyrus showed difficulty in written word construction to convey meaning.^10^ There have been two case reports on progressive changes in writing in patients with svPPA, displaying an increase in written output, including writing books or increased diary entries, though with a decrease in complexity over time.^11,12^ When comparing written to spoken spelling in a mixed group of participants with PPA, Henry *et al.*^13^ showed parallel changes in both modalities, suggesting proportional impairments deriving from core linguistic dysfunction in phonology and semantics. However, in a study of agrammatism in patients with nfvPPA compared to patients with primary progressive apraxia of speech (PPAOS), the nfvPPA group made more grammatical errors in speech than in writing relative to the PPAOS group.^14^

In this study, our goals—using a language-elicitation test (Western Aphasia Battery picnic scene)—were to compare the content and quantity of written communication between the three PPA variants and cognitively normal control participants, to compare written to spoken language in PPA patients, and to examine the relationships of written language output to the severity of aphasia and the severity of overall cognitive impairment. We developed an automated program that quantifies content units (CUs) and total units (words and word attempts) in transcribed spoken and written language samples, employing a predefined CU corpus.^15^ Based on our prior study of spoken language,^15^ we hypothesized a decrease in the total number of written CU and total units in participants with PPA compared to controls. In cases where syntax may be simplified, such as in participants with nfvPPA, we expected an increase in the ratio of CU to total units (CU/U Ratio). Prior studies comparing written to spoken language in healthy individuals (from primary school to graduate students), have shown a relative decrease in written output with a relative increase in the density of CU in written language compared to spoken.^16^ Therefore, we hypothesized an overall decrease in written compared to spoken CU in participants with PPA. Based on our clinical experience, we expected fewer task-irrelevant units, such as self-referential and tangential language, in written than in spoken language, especially in lvPPA and svPPA, leading to an increase in the CU/U Ratio. Also, based on our clinical experience, writing can sometimes be less impaired than speech in nfvPPA (especially in those with predominant apraxia of speech); in this case, the amount of CU in writing and in speech may be similar. Finally, we expected a proportional decrease in content in patients with relatively greater aphasia severity (Progressive Aphasia Severity Scale Sum of Boxes) and in those with greater cognitive and functional impairment (Clinical Dementia Rating scale Sum of Boxes).

## Materials and methods

### Participants

Seventy-five individuals diagnosed with PPA were included in this study, all of whom were recruited through the Massachusetts General Hospital (MGH) Frontotemporal Disorders Unit PPA programme. All participants (and their care partners for patients with PPA) gave written informed consent in accordance with guidelines established by the Mass General Brigham Healthcare System Institutional Review Boards who govern human subjects research at Massachusetts General Hospital. See **Table 1** for demographic and clinical data. All patients received a standard clinical evaluation comprising a structured history obtained from both patient and informant, comprehensive medical, neurological and psychiatric history and exams, neuropsychological and speech-language assessments and a clinical brain MRI that was visually inspected for (i) regional atrophy consistent with or not consistent with a given syndromic diagnosis; and (ii) other focal brain lesions or evidence of cerebrovascular disease. Clinical formulation was performed through consensus conference by our multidisciplinary team of neurologists, psychiatrists, neuropsychologists and speech and language pathologists.^17^ All patients included in this study met the diagnostic criteria for PPA, and all were able to be subclassified into one of the major subtypes with a clinical imaging-supported atrophy pattern.^2^ Furthermore, no patient had any other focal brain lesions or significant cerebrovascular disease (e.g. previous strokes, cerebral haemorrhages and meningiomas); none had major psychiatric illness not adequately treated; and all were native speakers of English. The cohort analysed here included 28 patients with nfvPPA, 30 patients with lvPPA and 17 patients with svPPA. Patients with mixed forms of aphasia or PPAOS were excluded.

**Table 1:** Research participant demographic and clinical data

	Controls spoken	Controls written	nfvPPA	lvPPA	svPPA	*P*
Sample size (*n*)	20	20	28	30	17	NA
Age (average ± SD)	62.9 ± 11.62	59.25 ± 9.72	71.21 ± 7.78^a^	69.83 ± 7^b^	66.82 ± 7.74^c^	<0.001
Sex (% female)	50%	65%	57.1%	46.6%	58.8%	0.736^d^
Years of education ± SD	15.8 ± 1.10	15.9 ± 1.65	15.8 ± 2.95	16.52 ± 2.44	16.44 ± 2.48	0.598^e^
Handedness (% right)	80%	95%	85.7%	76%	88.2%	0.106^d^
Years from symptom onset (average ± SD)	NA	NA	3.79 ± 2.06	4.5 ± 2.9	4.56 ± 1.12	0.325^e^
CDR CGS = 0 (*n*)^f^	NA	NA	7	5	1	0.517^d^
CDR CGS = 0.5 (*n*)	NA	NA	17	23	13	
CDR CGS = 1 (*n*)	NA	NA	2	2	2	
CDR SoB (average ± SD)	NA	NA	5.90 ± 4.35	5.48 ± 2.09	4.68 ± 1.86	0.124^e^
CDR language = 0.5 (*n*)^g^	NA	NA	18	15	9	0.144^d^
CDR language = 1 (*n*)	NA	NA	6	12	8	
CDR language = 2 (*n*)	NA	NA	4	1	0	
PASS SoB (average ± SD)	NA	NA	5.90 ± 4.35	5.48 ± 2.09	4.68 ± 1.86	0.532^e^

CDR CGS, Clinical Dementia Rating scale Calculated Global Score; CDR SoB, Clinical Dementia Rating scale Sum of Boxes; nfvPPA, nonfluent variant primary progressive aphasia; lvPPA, logopenic variant primary progressive aphasia; PASS SoB, Progressive Aphasia Severity Score Sum of Boxes; SD, standard deviation; svPPA, semantic variant primary progressive aphasia.

a Age not normally distributed, Kruskal–Wallis test *P* < 0.01when compared to controls (both written and spoken).

b Kruskal–Wallis test *P* < 0.001 when compared to controls (written) and *P* = 0.033 compared to controls (spoken).

c Kruskal–Wallis test *P* = 0.025 when compared to controls (written).

d Chi-squared test.

e Independent-samples Kruskal–Wallis test.

f Values not available for two patients with nfvPPA and one for svPPA.

g Values not available for two patients with lvPPA.

With respect to apraxia of speech and dysarthria in patients with nfvPPA, 17 (61%) patients were considered to have apraxia of speech, 5 (18%) had both apraxia of speech and dysarthria and 6 (21%) were considered to have neither. The severity of the articulatory impairments was quantified with the Progressive Aphasia Severity Scale articulation domain. Of the 28 patients with nfvPPA, 4 (14%) patients had normal articulation, 15 (54%) had questionable or very mild impairment, 3 (11%) had mild impairment, 4 (14%) had moderate impairment and 2 (7%) had severe impairment. Only one of these patients did not have intelligible speech and was therefore not considered for the written versus spoken analysis. Articulatory function, as expected, was minimally affected in patients with lvPPA (23 normal and 3 questionable or very mild) and svPPA (17 normal). Two patients with nfvPPA and three patients with lvPPA were not able to complete the written task. Of the two patients with nfvPPA, one patient reported that writing a description was ‘too difficult’, and the other patient was only able to write their name. The three patients with lvPPA who were unable to complete the task were agraphic (their writing impairment was too severe to score). Despite their ability to produce spoken samples, these patients could not be included in the analysis.

From the structured evaluations, each patient had a Clinical Dementia Rating (CDR) global score, a CDR Sum of Boxes (CDR SoB) score, a CDR Supplemental Language Box score and a Progressive Aphasia Severity Scale Sum of Boxes (PASS SoB) score.^18^ The CDR is a clinical dementia rating tool that reflects general aspects of cognition and activities of daily living.^19^ The PASS is a clinical instrument used to rate presence and severity of impairment in specific domains of speech and language.^18^

Spoken samples were also obtained from 20 healthy controls—with no self-reported history of neurologic or psychiatric disorders—from the Speech and Feeding Disorders Laboratory at MGH Institute of Health Professions. Written samples from 20 controls were obtained from the Amazon’s Mechanical Turk volunteers after they confirmed lack of any neurological or language-related abnormalities. Demographic features from these participants are presented in Table 1.

### Sample collection

Participants performed spoken and written descriptions of the Western Aphasia Battery—Revised (WAB-R) ‘Picnic Scene’ task. Spoken and written responses were obtained in a fixed order (spoken then written). Participants were instructed to produce descriptions in either modality using full sentences. Spoken samples were transcribed with the aid of Microsoft Word Version 16.57 dictation software, and manually corrected by either N.R. or S.J.H. (error rate was not quantified). Handwritten samples from patients with PPA were manually transcribed to text files by S.J.H., A.J., or I.H. (see the ‘Acknowledgements’ section). Spelling errors were manually corrected upon transcription and quantified separately. Written samples from control participants were typed and did not require any additional text processing. Both unintelligible spoken words and illegible written words were counted towards the total unit count, but not content unit count. The first author was blind to the participant status until the statistical analysis.

### Definitions

Quantified terms are defined as follows:

- Content unit (CU): ‘Correct information units are words that are intelligible in context, accurate in relation to the picture or topic, and relevant to and informative about the content of the picture or the topic. Words do not have to be used in a grammatically correct manner to be included in the correct information count.’^20^ Following Berube *et al.*,^21^ the CU dictionary was compiled from CUs that were each mentioned in spoken descriptions by at least three healthy controls. The 64-CU dictionary used was previously published by Gallée *et al*.^15^ Each CU is only counted once, regardless of how many times it is mentioned in a sample. The previously published CU dictionary also grouped morphological variants within one single CU.^15^ For example, the nouns ‘girl’ and ‘daughter’ are listed as CU #12. Therefore, if one participant used both words (girl and daughter), they would only be counted as one CU. We followed the same dictionary for consistency.
- Unambiguous CU: Refers to a unique object/entity, action, or property of a specific object/entity in the picture.^15^ The term used can only apply to a single feature within the scene. For example, there is only one dog in the scene, therefore, ‘dog’ is an unambiguous CU.
- Ambiguous CU: Does not refer to a unique aspect of the picture.^15^ The term in isolation can correspond to more than one feature within the scene. The term will be labelled as ambiguous even if context suggests a particular CU. For example, in the picnic scene, where both the dog and the boy are running, the term ‘running’ is always an ambiguous CU.
- Self-referential: Any first-person pronoun—I, we and us.
- Unit (U): Total count of every word, non-word, false start. False starts were defined as ‘Phonemic clusters followed by self-corrections or rerouting.’^15^ Contractions were counted as two units. For example, ‘they’re’ = 2 units.
- CU/unit ratio (CU/U Ratio): Total CU (repeated CU only counted once) divided by total units. In other publications, this corresponds to the term ‘informativeness.’^15^ However, the term informativeness may be misleading. A higher value does not always reflect a language sample that conveys greater meaning, as it not only depends on the number of CUs (numerator) but also the total number of units (denominator). For example, a list of CUs without basic sentence structure will have a higher CU/U Ratio but convey less meaning without words that capture relationships between the CUs.

### Program

We used Quantitext, a text analysis toolbox we developed in the Frontotemporal Disorders Unit of MGH, to automatically produce a set of quantitative language metrics. The goal of developing this package is to increase the precision and objectivity of language assessments while reducing human labour.^22^ The toolbox uses a number of natural language processing toolkits and software such as Stanford Parser,^23^ spaCy,^24^ as well as text analysis libraries in R. Quantitext receives typed or transcribed language samples (in the case of spoken or handwritten samples) as input and generates as outputs in a number of metrics such as sentence length, log word frequency, log syntax frequency, content units, total units, efficiency of lexical and syntactic items and part-of-speech tags. To specify content units, the toolbox first generates a python dictionary using a predefined set of words as previously described^15^ and then uses this dictionary to automatically identify all content units in new texts that it receives as input. Lemmatization of words in the program allows for the identification of words in various morphology (e.g. run, ran, running, etc.). The program then counts the content units and all units in the language sample of each participant. To measure the program’s validity, each sample was then manually annotated to check for omissions and proper counting.

### Statistical analysis

Statistical analysis was performed with IBM SPSS Statistics Version 28.0.0.0 and Prism 9 for macOS Version 9.3.0 (345). Our program’s validity was assessed with a Pearson bivariate correlation analysis. Ordinal and nominal values were compared between groups using Chi-square. Supplementary Table 2 shows the Shapiro–Wilk test for normality distribution per group and type of measurement. Highlighted in red are those groups/measurements without a normal distribution. Despite 9 out of 16 of the measured samples/groups not having a normal distribution, given the acceptable sample size per group, we opted for parametric tests (ANOVA) as the best form of analysis to measure group differences. Given the size of the differences between and within groups, there is a low risk for type 1 error. When significant differences were present, *post hoc* analysis was performed with Tukey’s *post hoc* multiple comparisons procedure. When comparing written to spoken language between the three main PPA subtypes, a one-between (PPA subtypes)–one-within (written versus spoken) ANOVA was used. Within participant analysis was done exclusively for written and spoken samples collected within 1 month of each other. However, most samples were collected during the same testing session or within days from each other. The only exception was one patient with nfvPPA with a one-month difference between the modalities. Bivariate Pearson correlation analysis was performed correlating written and spoken language measurements to clinical scales. Results were considered statistically significant when *P* < 0.05, and trends are reported when *P* < 0.1.

## Results

### Participants

The clinical and demographic characteristics of the participants are shown in Table 1. The average age of patients in the three PPA subtypes was higher than that of control participants in the written sample group. The average age in the nfvPPA and lvPPA groups was higher than that of control participants in the spoken sample group. Otherwise, there were no significant differences between control participants and participants with the three different PPA subtypes in sex distribution, years of education and handedness. There were no significant differences between the three PPA subtypes in years from symptom onset, average CDR scores, CDR SoB, CDR Supplemental Language Box and PASS SoB.

### Validity and efficiency of automated language analysis program

On average, it took the program 2.4 s per participant to parse the text, assign ambiguous, unambiguous and self-reference labels to related words and provide the total count of each type of CU and all units. Manual text analysis to provide similar metrics takes ∼20 to 30 minutes per participant. The automated program identifies total units with a validity of 100% and CUs with a validity of 99.7% (CI 99.5–99.8, *P* < 0.001) compared to manual annotation of the samples. In the automated analysis of spoken samples of healthy controls, 66.7% of the errors were related to missing unambiguous CU, 33.3% to ambiguous CU, with no errors in counting references to self. In the analysis of the written samples of healthy controls, 20% of the errors of the program were related to missing unambiguous CU, 80% to ambiguous CU, with no errors in counting references to self. In the spoken samples of the patients, 81.8% of the errors of the program were related to counting unambiguous CU, 18.2% to ambiguous CU, with no errors in counting references to self. Finally, in the written samples of the patients, 54.8% of the errors of the program were related to counting unambiguous CU, 22.6% to ambiguous CU and 22.6% to references to self. All counting errors were due to parsing mistakes of the automatic parser, resulting in the assignment of the wrong part-of-speech to words. These errors were manually corrected prior to statistical analysis.

### Functional written communication in PPA

**Figure 1:**
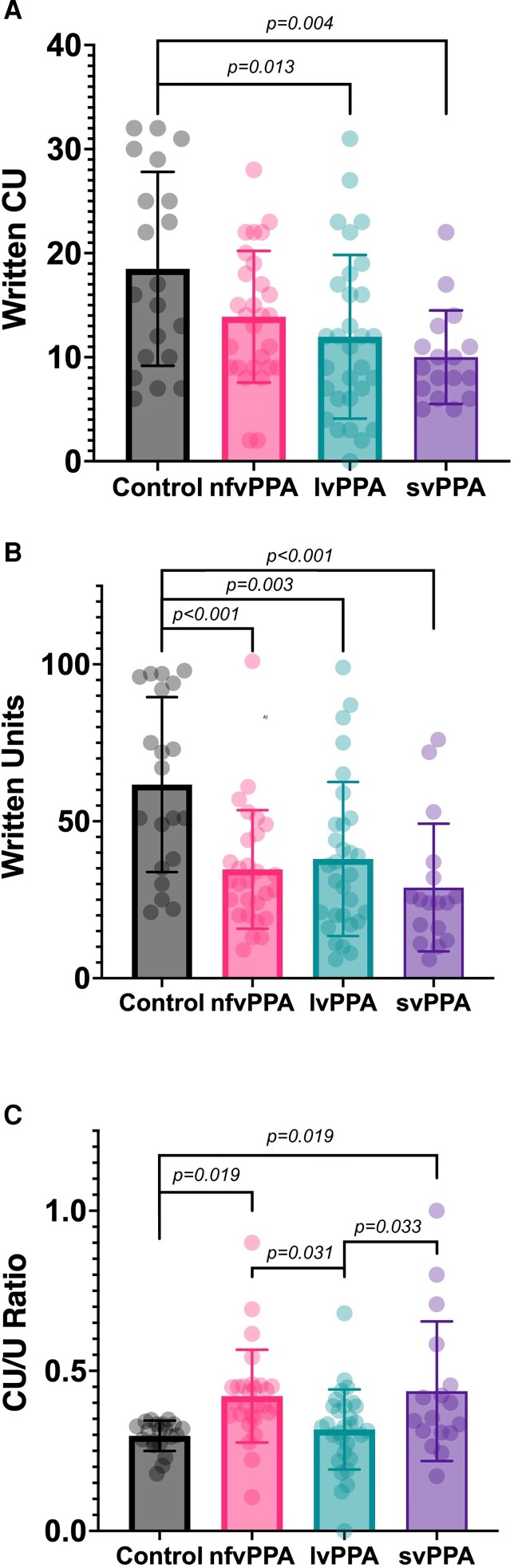
**Comparison of written language between participants with PPA and controls.** Graphs illustrate written content units (CUs) (**A**), total units (U) (**B**) and CU/U Ratio (**C**) in controls and the three PPA variants. Error bars indicate 1 SD. Statistically significant differences indicated by lines correspond to one-way ANOVA with Tukey’s *post hoc* tests.

As expected and shown in Fig. 1 and Table 2, all patients with PPA wrote fewer total units than controls (*F*(3,91) = [7.778], *P* < 0.001), with large effect sizes (Cohen’s *d* = 1.14 for nfvPPA, 0.90 for lvPPA and 1.34 for svPPA). There were no differences between the three PPA variant groups. As for CUs, patients with lvPPA and svPPA wrote fewer than controls (one-way ANOVA *F*(3,91) = [4.897], *P* = 0.003; Tukey’s *post hoc* test *P* = 0.013 for lvPPA and 0.004 for svPPA), also large effect sizes (Cohen’s *d* = 0.76 for lvPPA and 1.16 for svPPA). The average number of CUs written by the nfvPPA group was lower than controls, but this was not a statistically significant difference (Tukey’s *post hoc* test *P* = 0.14). There were no differences between the three PPA variant groups in written CUs. The CU/U Ratio was higher in nfvPPA and svPPA than controls (*F*(3,91) = [5.592], *P* = 0.019), large effect sizes (Cohen’s *d* = 1.14 for nfvPPA and 0.88 for svPPA), but no difference between lvPPA patients and controls (*P* = 0.962). The CU/U Ratio was higher in nfvPPA and svPPA than lvPPA (Tukey’s *post hoc* test *P* = 0.031 for nfvPPA and *P* = 0.033 for svPPA), moderate effect sizes (Cohen’s *d* = 0.77 for nfvPPA and 0.68 for svPPA). When age was added as a covariate in an analysis of covariance, it did not enter any models (all *P*-values of >0.1).

**Table 2:** Measurements of written language

Group	CU (average ± SD)	Units (average ± SD)	CU/U Ratio (average ± SD)	*n*
Control	18.5 ± 9.32	61.70 ± 27.86	0.30 ± 0.05	20
All PPA	12.24 ± 6.74	34.68 ± 21.63	0.38 ± 0.16	75
nfvPPA	13.89 ± 6.33	34.64 ± 18.87^a^	0.42 ± 0.14^b^	28
lvPPA	11.97 ± 7.87^a^	38.00 ± 24.54^a^	0.32 ± 0.12	30
svPPA	10.00 ± 4.50^a^	28.88 ± 20.36^a^	0.44 ± 0.22^b^	17

CUs, content units; SD, standard deviation; CU/U Ratio, content unit/unit ratio; PPA, primary progressive aphasia; nfvPPA, nonfluent variant primary progressive aphasia; lvPPA, logopenic variant primary progressive aphasia; svPPA, semantic variant primary progressive aphasia.

a Significantly decreased when compared to control participants (refer to Fig. 1 for specific *P*-values).

b Significantly decreased when compared to control participants and participants with lvPPA (refer to Fig. 1 for specific *P*-values).

We obtained the mean and standard deviation for the written CU/U Ratio in all samples (0.364 ± 0.151) and qualitatively examined the samples of patients with CU/U Ratios 1 SD below (0.213) and 1 SD above the mean (0.516). There were nine participants with a CU/U Ratio with at least 1 SD below the mean. Those nine participants comprised two control participants, one patient with nfvPPA, one with svPPA and five with lvPPA. These samples either had a very small number of CUs, or had a larger number of tangential, vague and self-referential units that did not add content to the description. There were also nine participants with a CU/U Ratio with at least 1 SD above the mean. Those nine participants comprised four patients with nfvPPA, four with svPPA and one with lvPPA. These descriptions tended to be short, and in some cases, consisted of a list of CUs with a very simple or absent sentence structure. For example, in the phrase: ‘Woman pouring drink’, every unit corresponds to a CU.

### Comparison of written and spoken languages in PPA patients

**Figure 2:**
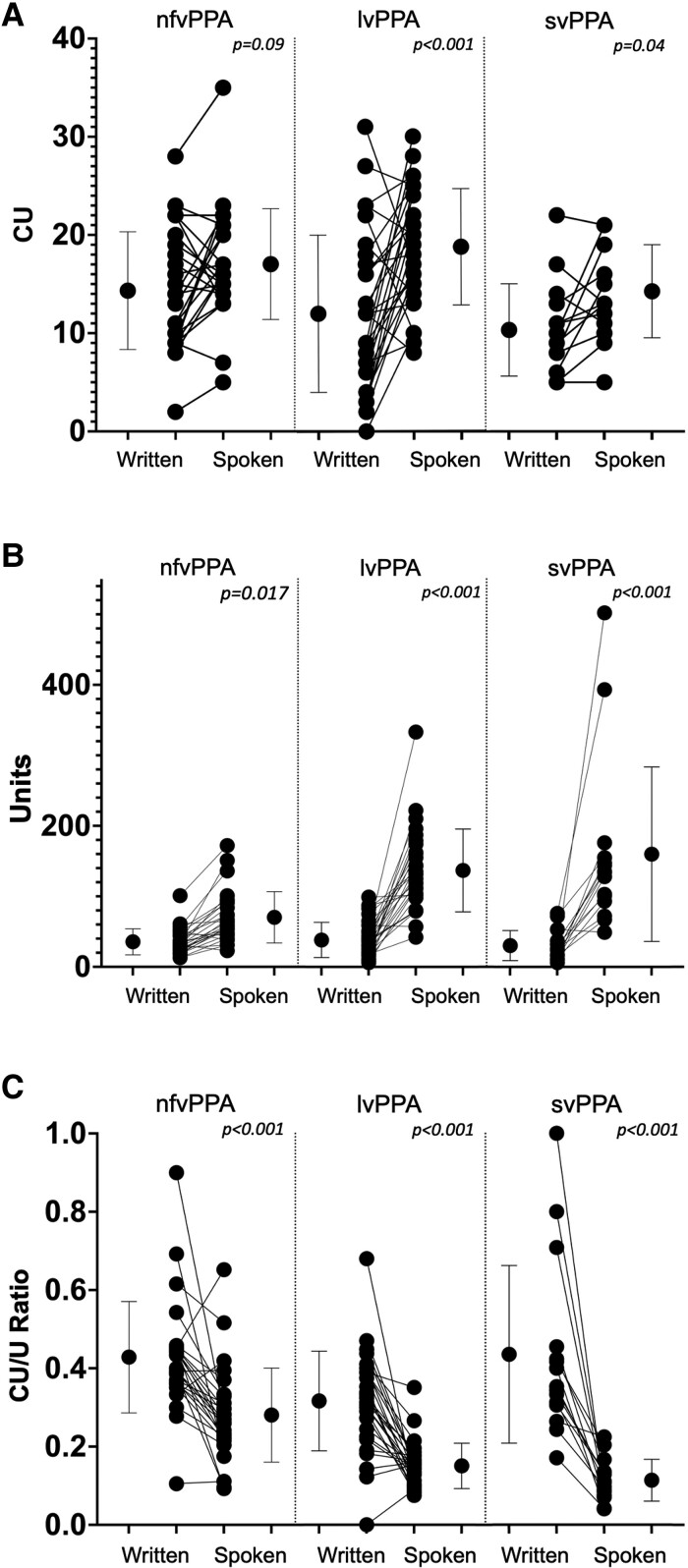
**Comparison of written and spoken languages within participants with PPA.** Each graph shows the mean and 1 SD for the group of participants on the left (written) and right (spoken) of each panel, with individual datapoints from the same participant connected by a line in the *middle*. Data from each of the PPA variants are displayed separately. The rows of graphs show content units (CUs) (**A**), total units (**B**) and CU/U Ratio (**C**). The *P*-values at the *top* of each panel correspond to Tukey’s HSD *post hoc* contrasts, in a two-way one-within/one-between ANOVA comparing the written to spoken modality between each PPA subtype.

Fig. 2 and Table 3 show comparisons of written versus spoken language in the three PPA subtypes. Examining the three PPA variants, the overall number of CUs was less in written versus spoken language (*F*(1,66) = [24.17], *P* < 0.001). Tukey’s *post hoc* analysis showed that participants with lvPPA (*P* < 0.001) and svPPA (*P* = 0.042) produced fewer written CUs than spoken CUs, but this effect was only present as a trend in participants with nfvPPA (*P* = 0.09). The overall number of total units was less in written versus spoken language in all subtypes (*F*(1,66) = [96.82], *P* < 0.001). The CU/U Ratio was greater in written than spoken language for all PPA subtypes (*F*(1,66) = [93.74], *P* < 0.001).

**Table 3:** Comparison in language measures according to modality in the three main PPA subtypes

	Modality/CI	nfvPPA	lvPPA	svPPA
Group *n*		26	28	15
Language measures per modality				
CU	Written (average ± SD)	14.35 ± 6.11	12.00 ± 8.15	10.33 ± 4.7
	Spoken (average ± SD)	16.85 ± 5.67	19.14 ± 5.64	14.27 ± 4.74
	95%CI^a^	−5.41 to 0.41	−9.92 to −4.37	−7.76 to −0.11
Units	Written (average ± SD)	35.58 ± 18.92	38.25 ± 25.41	30.20 ± 21.23
	Spoken (average ± SD)	69.73 ± 37.01	138.96 ± 58.69	159.93 ± 123.70
	95%CI^a^	−62.12 to −6.19	−127.74 to −73.69	−166.55 to −92.92
CU/U Ratio	Written (average ± SD)	0.43 ± 0.14	0.32 ± 0.13	0.44 ± 0.23
	Spoken (average ± SD)	0.28 ± 0.12	0.15 ± 0.06	0.11 ± 0.05
	95%CI^a^	0.081 to 0.22	0.10 to 0.23	0.23 to 0.41

CUs, content units; SD, standard deviation; CU/U Ratio, content unit/unit ratio; nfvPPA, nonfluent variant primary progressive aphasia; lvPPA, logopenic variant primary progressive aphasia; svPPA, semantic variant primary progressive aphasia.

a CI, Tukey’s HSD 95% confidence interval: comparing the written versus spoken modality within PPA subtype.

At the individual participant level, although the total number of total units was less in written compared to spoken language in nearly every single patient with PPA (92.75% of participants), this was not so universally true for CUs. Written CUs were fewer than spoken CUs in 61.54% of participants with nfvPPA, 82.24% of those with lvPPA and 80.00% of those with svPPA. The CU/U Ratio was greater in written than spoken language for 84.62% of participants with nfvPPA, 89.29% of those with lvPPA and 100% of those with svPPA. The differences in these values, illustrated in Fig. 2, reflect the intra-group variability in written versus spoken language production within and between different PPA subtypes.

**Figure 3:**
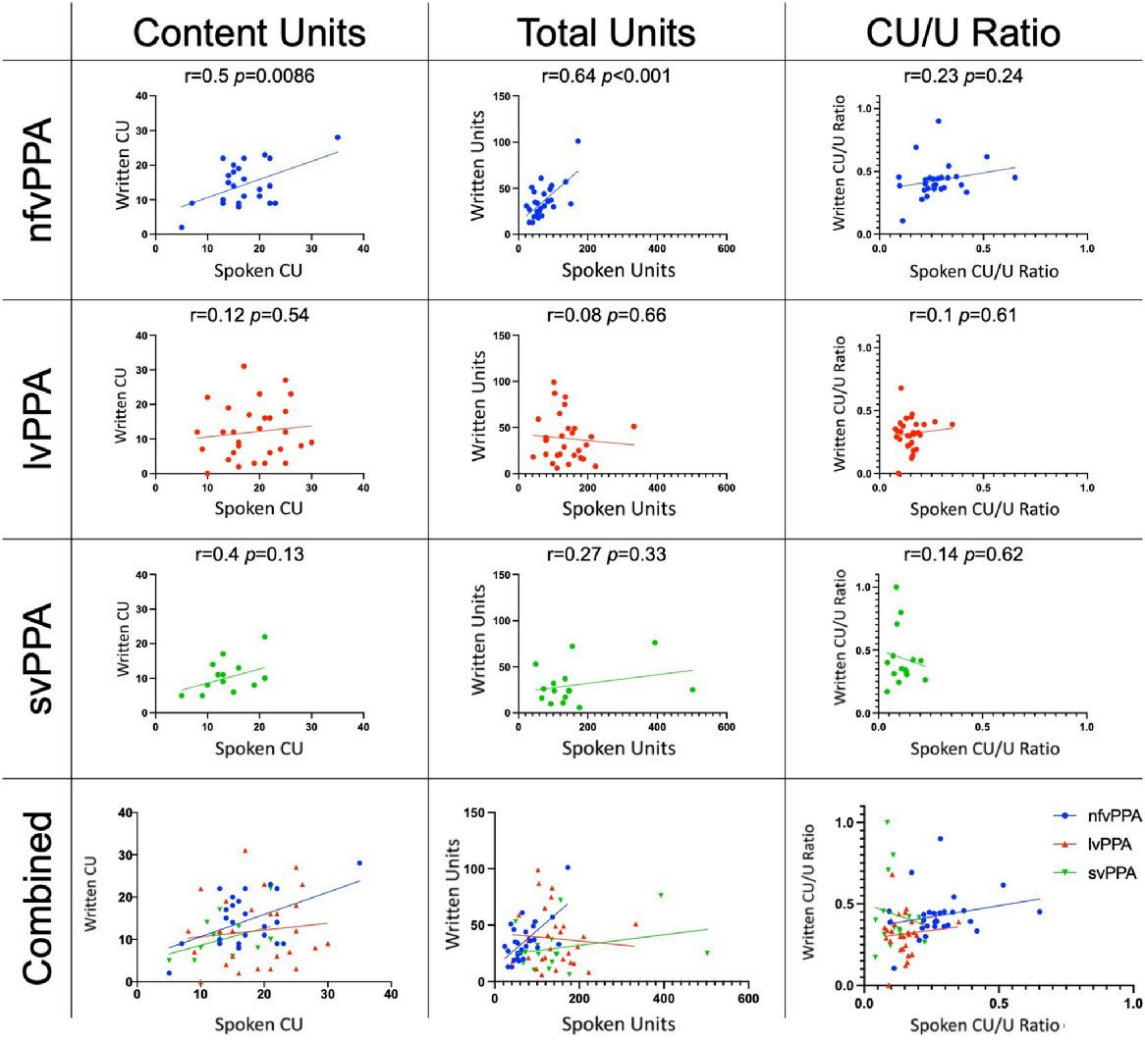
**Scatterplots comparing spoken and written CUs, total units and CU/U Ratio.** In all graphs, written language is on the *y*-axis, and spoken language is on the *x*-axis. The left column shows CU, the *middle* column shows total units and the right column shows CU/U Ratio. The rows separate the three PPA variants, with the *bottom* row showing the three variants combined. Statistics included refer to Bivariate Pearson correlation analysis.

As illustrated in Fig. 3, we performed correlation analysis between the spoken and written modalities for CU, total units and the CU/U Ratio in each PPA variant. In the nfvPPA group only, there is a significant correlation between the number of spoken CU and written CU (*r* = 0.5, *P* = 0.0086) and between the number of spoken and written total units (*r* = 0.64, *P* < 0.001). There is a non-significant correlation between spoken and written CUs in svPPA (*r* = 0.4, *P* = 0.13). There was no significant correlation in written versus spoken CU (*r* = 0.12, *P* = 0.54) and units (*r* = 0.08, *P* = 0.66) in lvPPA, or units in svPPA (*r* = 0.27, *P* = 0.33). There was also no significant correlation between spoken and written CU/U Ratio in any of the subtypes (all *P*-values of >0.24).

The scatterplots show several features worth highlighting: (i) in the case of spoken versus written CU, especially in the case of lvPPA, there are some participants who say many more CU than they write, and vice versa; and (ii) for the CU/U Ratio, patients with svPPA exhibit an extreme distribution with an almost vertical plane, reflecting much greater variability in written CU/U Ratio compared to spoken CU/U Ratio; lvPPA patients show a somewhat similar but less extreme effect. In both cases, the pattern appears to be due to the relative absence of empty language in the written modality. This is in contrast with patients with nfvPPA, where participants have a relatively higher CU/U Ratio in both writing and speech.

We also examined the ambiguity of CUs, comparing written to spoken language samples (Table 4). In the spoken modality, there were significant differences in the proportion of unambiguous and ambiguous CUs to total CU, specifically between patients with nfvPPA and controls, as well as between nfvPPA and the other two PPA subtypes (one-way ANOVA *F*(3,94) = [10.281], *P* < 0.001), with a preference towards unambiguous CU. Though not statistically significant, patients with svPPA have a relative predilection for ambiguous CU (relatively fewer unambiguous CU as seen in Table 4). Despite a similar general pattern observed, when compared to the spoken modality, there were no significant differences in the proportion of written unambiguous and ambiguous CUs to total CU between PPA and controls, or between the different PPA subtypes (one-way ANOVA *F*(3,94) = [1.197], *P* = 0.316). This lack of significant differences may be explained by increased variability in the written modality when compared to the spoken modality. However, a three-between groups ANOVA showed that there were no differences in the distribution of ambiguous and unambiguous CUs between the three PPA subtypes and controls, when comparing the written to the spoken modality (*F*(3,342) = [0.54], *P* = 0.65). As above, when age was added as a covariate in an analysis of covariance, it did not enter any models (*P* > 0.1).

**Table 4:** Unambiguous and ambiguous contents in spoken and written samples in control participants and in the three main PPA subtypes

	Measure	Control	nfvPPA	lvPPA	svPPA
Spoken	Unambiguous CU/total CU Average ratio ± SD (*n*)	0.82 ± 0.05 (20)	0.88 ± 0.06 (27)^a^	0.81 ± 0.06 (30)^b^	0.76 ± 0.1 (17)^c^
	Self-referential pronouns Average ± SD	1.55 ± 1.5	0.96 ± 2.33	2.80 ± 2.71	7.29 ± 9.74^d^
Written	Unambiguous CU/total CU Average ratio ± SD (*n*)	0.81 ± 0.07 (20)	0.86 ± 0.1 (28)	0.83 ± 0.1 (29)	0.80 ± 0.1 (17)
	Self-referential pronouns Average ± SD	0.15 ± 0.37	0.07 ± 0.38	0.27 ± 0.78	0.00

CUs, content units; SD, standard deviation; CU, content unit; nfvPPA, nonfluent variant primary progressive aphasia; lvPPA, logopenic variant primary progressive aphasia; svPPA, semantic variant primary progressive aphasia.

a One-way ANOVA, Tukey’s HSD *post hoc* contrast *P* = 0.019 compared to spoken control.

b One-way ANOVA, Tukey’s HSD *post hoc P* = 0.003 compared to spoken nfvPPA.

c One-way ANOVA, Tukey’s HSD *post hoc P* < 0.001 compared to spoken nfvPPA.

d One-way ANOVA, Tukey’s HSD *post hoc P* < 0.01 compared to spoken control, nfvPPA and lvPPA.

Finally, based on our prior observations of self-referential speech in svPPA,^15^ we examined self-referential pronouns in writing. As seen in Table 4, there were no differences in written self-referential pronouns between the three PPA subtypes and controls (*F*(3,91) = [1.184], *P* = 0.32). Participants with svPPA had more spoken self-referential pronouns than controls or participants with nfvPPA or lvPPA (*F*(3,91) = [7.408], *P* < 0.001).

### Relationships between language measurements and severity of clinical impairment in PPA

**Figure 4:**
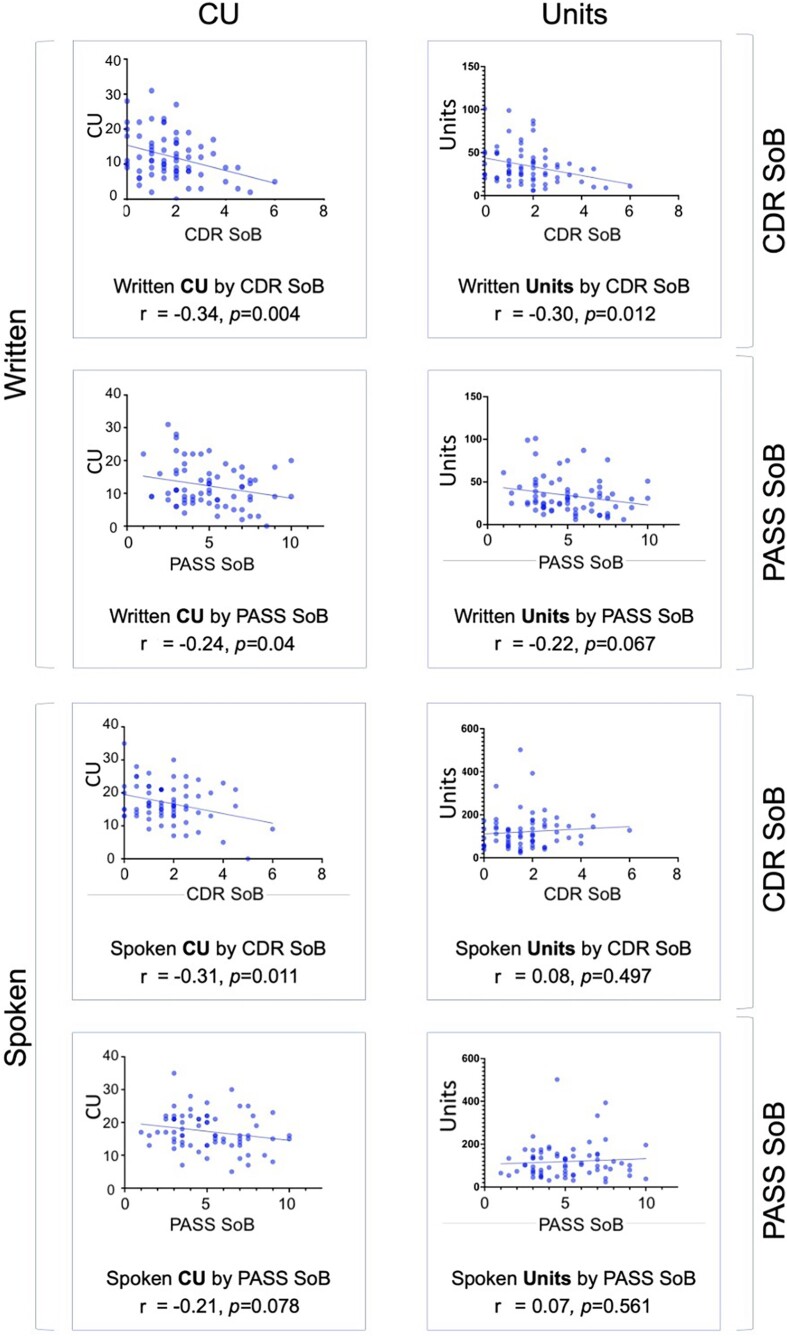
**Correlation analysis of written and spoken CUs and total units from all participants with PPA against their respective clinical rating scores.** The left column corresponds to content units (CUs) and the right to total units. The *top* four boxes correspond to the written modality and the *bottom* four to spoken. Each modality is further subdivided into correlation with the Clinical Dementia Rating scale Sum of Boxes (CDR SoB) or the Progressive Aphasia Severity Scale Sum of Boxes (PASS SoB) as shown on the right. Statistics shown correspond to Bivariate Pearson correlation analysis.

Although the patients in this sample had mild-to-moderate degrees of aphasia and only prodromal to mild dementia, we examined relationships between written language metrics of interest and aphasia severity (PASS SoB) and dementia severity (CDR SoB). As seen in Fig. 4, correlation analysis showed small effect size relationships indicating that written CUs are reduced in patients with greater dementia severity (CDR SoB *r* = −0.34, *P* = 0.004) and greater aphasia severity (PASS SoB *r* = −0.24, *P* = 0.04). A reduction in spoken CU was also observed with greater dementia severity (CDR SoB *r* = −0.31, *P* = 0.011) with a trend towards the same relationship with greater PASS SoB score (*r* = −0.21, *P* = 0.078). Similarly, for total written units, a decrease was observed with increasing CDR SoB (*r* = −0.30, *P* = 0.012) and a trend with PASS SoB (*r* = −0.22, *P* = 0.067). No significant correlation was observed between spoken total units and CDR SoB (*r* = 0.08, *P* = 0.497) or PASS SoB (*r* = 0.07, *P* = 0.561). Regarding the CU/U Ratio, a significant correlation was only observed between spoken CU/U Ratio and CDR SoB (*r* = 0.29, *P* = 0.018), but not with PASS SoB (*r* = 0.08, *P* = 0.527) or between written CU/U Ratio and CDR SoB (*r* = 0.03, *P* = 0.753) or PASS SoB (*r* = 0.04, *P* = 0.718). This study was not powered to examine these relationships in each PPA variant.

## Discussion

In this work, we investigated the density of content of picture-elicited written language samples in patients with PPA and control participants using an automated computational analytic method. We also compared the density of content of written language samples to spoken language samples elicited using the same task. We showed that despite a decrease in written CUs and units in participants with PPA, there was a relative increase in the density of content in written language compared to spoken language, especially in patients with lvPPA and svPPA due to a lesser amount of ‘empty’ language. In patients with nfvPPA, written language was frequently ‘telegraphic’ as previously described.^6,25^ We observed similar reductions in the generation of ambiguous CUs between writing and speech in nfvPPA. Finally, we showed that CUs decrease proportionally to greater severity of aphasia or cognitive/functional impairment.

In the analysis of written language in PPA, spelling has been more thoroughly explored,^26-30^ but other elements of written language have received less attention. Written language in nfvPPA has been described as ‘telegraphic’^6,25^ that, as hypothesized, we found here as well: nfvPPA patients use content words over non-content words, ultimately leading to a higher CU/U Ratio. In contrast, Graham *et al.*^6^ did not find higher ‘information units per word’ in patients with nfvPPA compared to controls, which may be due to controls in our study writing or saying fewer CU. This in turn could be due to a difference in instructions, our ‘describe this picture using full sentences’ as opposed to Graham’s ‘Tell/write everything you see going on in this picture’. Lastly, we extend prior work to also demonstrate a similar increase in written CU density in svPPA, when compared to controls, and in all PPA subtypes when comparing writing to the patients’ own spoken language.

The ‘primary systems’ hypothesis predicts that parallel changes are expected to occur in spoken and written languages.^31^ This hypothesis has been supported in spelling in PPA,^13^ but to our knowledge, not using other measures of written versus spoken language. We observed similarities in the pattern of ambiguous and unambiguous CUs in both written and spoken languages in all PPA variants, supportive of the primary systems hypothesis. The significant correlation between written and spoken CUs and units in patients with nfvPPA would be supportive of the hypothesis. However, the same correlation was not observed in the logopenic and semantic variants, which limits its generalizability. When comparing CUs, units and CU density (CU/U Ratio), we observed a disproportionate decrease in total units relative to CU between written and spoken samples, predominantly in lvPPA and svPPA (but also in nfvPPA), leading to a relative increase in the CU/U Ratio. A similar increase in ‘information units per word’ in written compared to spoken descriptions had been previously reported by Graham *et al.*^6^ in patients with nfvPPA and controls.

The difference in written versus spoken language has not been previously published in lvPPA or svPPA. Given that Alzheimer’s disease pathology is most often the underlying aetiology for lvPPA, and their shared neuroanatomic distribution affecting the temporo-parietal junction,^2,32^ previous reports on writing in lvPPA have provided comparisons with patients with MCI or Alzheimer’s disease dementia, demonstrating higher verb use in patients with lvPPA.^9^ Also, studies comparing written to spoken language in Alzheimer’s disease dementia have shown differences in the information pattern between both modalities, with written descriptions being as informative, but shorter and syntactically simplified compared to spoken descriptions.^33^ The latter finding has some overlap with our own, in that written descriptions were shorter and simplified compared to spoken in patients with lvPPA. However, CUs were fewer in the written than the spoken modality, which may argue against equivalent informativeness despite the lack of significant empty language in writing compared to speech.

As previously mentioned, Ravid and Berman^16^ have showed a relative decrease in written output compared to spoken, as well as a relative increase in the density of CU in healthy individuals. In patients with PPA, we observed within-group differences, with some patients writing more than they spoke and vice versa. One could hypothesize that within control participants, the direction of change would be more consistent, with the majority having greater spoken than written output, but this requires further study. It is also interesting how, between both control groups, the ratio of content units to units followed the same pattern as within the PPA groups, with a higher value in written than spoken samples (though only a trend statistically, Supplementary Table 1 and Supplementary Fig. 1).

Studies of writing in svPPA include reports of increased production and creativity in writing in three patients with svPPA,^34^ as well as a longitudinal writing analysis in two patients with svPPA.^11,12^ Changes in writing in these cases may be attributed to changes in behaviour commonly observed in svPPA.^2^ Heitkamp *et al.*^11^ studied longitudinally the personal diary of a patient with svPPA, reporting an increase in the length of the diary entries with a concomitant decrease in vocabulary over time. Also, they noticed an increase in ambiguity (which we observed in participants with svPPA as well), a decrease in the variety of vocabulary, an increase in type-token ratio (which may be comparable to the increase in CU/U Ratio we report here) and simplified syntax at later stages.^11^ In the second longitudinal analysis of written text in a patient with svPPA, Hwang *et al.*^12^ also showed a decrease in lexical sophistication and an increase in ambiguity, the latter similar to our findings.

One goal of either spoken or written descriptions is to convey information effectively and efficiently, even if a language or other cognitive impairment interferes with the process. Ravid *et al.*^16^ showed that information density depends on the language modality, with significantly more ancillary (non-descriptive) material found in spoken when compared to written narratives. These findings may reflect more rapid ‘online’ processing in spoken narratives. In written narratives, there may be a higher cognitive demand, but also more planning and monitoring (‘offline production’).^16^ The manner in which a patient with PPA maximizes their ability to convey information is expected to depend on the nature of their language impairment and the language modality being employed. For example, given that participants with PPA have an overall decreased output in written language, they may prioritize words that will be more informative, at the expense of ancillary material and repairs.^16,35^ We recently reported this type of trade-off between word complexity and syntax in spoken language in PPA.^36^ In spoken language in the case of lvPPA and svPPA, specific word retrieval is challenged despite relatively preserved fluency in conversation. Participants with these subtypes may therefore say more overall, including circumlocutions and repairs, with the overall objective of conveying as much information as possible, at the expense of a decrease in information density. The increase in CU/U Ratios in written compared to spoken language may also reflect the optimization of investing a higher cognitive effort in fewer yet more informative language units.

It is important to explore the difference between the term informativeness and the CU/U Ratio. The term informativeness has been used to describe the communication of CUs relative to all words in speech,^15,21^ and calculated with the same equation we used here as the CU/U Ratio. We chose not to use the term informativeness here because, even in patients with relatively mild PPA, some individuals simply wrote a list of CUs without full sentence structure (despite the task instructions to write in full sentences). This occurred with lower frequency in speech samples, and usually in more impaired patients. As such, the higher CU/U Ratio reflected the high percentage of content words. While a list of nouns or verbs does provide content, it lacks informativeness about the relationship between the items. We found this to be the case only with some of the more severely impaired patients, whose writing samples contain few or no grammatical words, or whose samples are very short. Future investigation would be helpful to determine if the rating system presented here has the greatest utility in patients at a mild-to-moderate severity level of impairment, and less so for more severely impaired patients. Nevertheless, the CU/U Ratio helps to identify outliers whose language can be further examined quantitatively or qualitatively, as we explored in our results.

To the best of our knowledge, there is no other automated program that is equivalent to the code described in this manuscript. The program is adaptable to pictures other than the picnic scene by simply changing the dictionary of content units and has a validity of 99.7% in identifying CU and 100% in identifying total units. The program is also time efficient as it reduces the duration of text analysis of each participant from 20–30 minutes by manual raters to 2.5 s. As the program currently stands, it still requires transcription of spoken or handwritten samples, time that was not quantified in this research. However, if typed descriptions by participants are included in the future, this would eliminate the only current manual task in the process.

Clarke *et al.*^37^ thoroughly explored the multiple technologies that have been developed for automated language analysis and their role in MCI, Alzheimer’s disease and dementia with Lewy bodies. However, such technologies have scarcely been tested in PPA. Two such examples include the automated analysis of spelling errors^38^ and the automated analysis of two written works in a patient with svPPA.^12^ In-depth analysis of written language can be time consuming, and therefore is not routinely incorporated into clinical practice. As many societies become more familiarized with technologies, from typing on a smartphone to typing on a computer, tools that analyse written language can rapidly provide objective and likely clinically relevant information.

In this work, we also determined whether the reduction in written and spoken CUs and total units was merely a reflection of general cognition in participants with PPA. The mild-to-moderate correlation observed between the CDR SoB and written CU and units, as well as spoken CU, shows how these measures are only a partial reflection of general cognition, though may act as an indirect marker. Interestingly, Mazzeo *et al.*^39^ recently showed how written phrases under dictation can act as a predictor for total loss of speech in AD-related PPA.

The main limitation of this study is the fact that two separate control groups were used, one for spoken samples and one for written. This prevented a within-group analysis comparing language modalities from being performed in the control group. Also, regarding sample collection, the fixed order in spoken followed by written sample production (not counterbalanced) may introduce a practice effect. However, we believe that any influence of the task order on performance would be minimal and remain constant between groups. An important study design weakness (confound) is that the control group for written samples performed typed descriptions of the picnic scene, as opposed to the handwritten descriptions by participants with PPA. Based on time and effort required to type compared to handwrite, we would expect typed descriptions to be longer than handwritten descriptions. This may accentuate the difference in the total CU and units when comparing control participants to participants with PPA; however, the reduction observed in the participants with PPA is still more likely to be due to word retrieval difficulties. We did not observe a significant difference between the CU/U Ratio written and spoken samples in the control groups further supporting the validity of the data as representations of healthy human language production (Supplementary Table 1 and Supplementary Fig. 1). Samples that were not typed were manually transcribed and reliability not assessed, which may also introduce error into the study. In the future, using only typed samples would remove this variable. Next, as stated in the participants section, the average age of patients in the three PPA subtypes was significantly higher than the ‘written’ controls, and of the nfvPPA and lvPPA groups than the ‘spoken’ controls. This difference may affect the comparison of written samples between control participants and patients with PPA (units, content units and ratio), as well as spoken and written ambiguous/unambiguous CUs. However, as supported by analysis of covariance and given the magnitude of the differences observed, we believe that the findings reported here reflect the patients’ aphasia and are not simply a reflection of the control participants’ younger age. Another limitation is the high level of education in our samples, which may limit the generalizability of our findings. Finally, the correlation analysis with clinical rating scales was performed by pooling the samples of all participants with PPA, given that the study was not powered to analyse these variables between PPA variants separately.

There is much more to investigate in the writing of patients with PPA. Future studies may include analyses of specific elements of written language, such as grammar, word familiarity, syntax, verb and noun proportions and their comparison to speech. The reported changes in language should also be evaluated longitudinally. We would expect to find stronger relationships between written and spoken CUs and language/cognitive scales if they were tracked over time within individual patients.^8,11,12,40^ Future studies may also examine naturalistic production of written language, such as analysing text messages or emails, which may more closely reflect a patient’s day-to-day functional communication. It would also be relevant to study whether similar changes to those described in this manuscript occur in languages other than English. Lastly, it would be valuable to examine the neural correlates of written language impairments using neuroimaging.

## Supplementary Material

fcad202_Supplementary_Data

## Data Availability

The code for the program along with written samples from healthy controls as described in this manuscript is available in https://dataverse.harvard.edu/dataset.xhtml?persistentId=doi:10.7910/DVN/GWZRRO. The data that support the findings of this study are available on request from the corresponding author. The data are not publicly available due to the presence of information that could compromise the privacy of research participants. Derived data supporting the findings of this study are available from the corresponding author on request.
