# Supplementary material for: Automated analysis of written language in the three variants of primary progressive aphasia
Source: Brain Commun. 2023 Jul 20;5(4):fcad202. doi: 10.1093/braincomms/fcad202 (PMC10396070; doi:10.1093/braincomms/fcad202)
Supplement: fcad202_Supplementary_Data [file fcad202_supplementary_data.pdf]

Supplementary Material

Supplementary Table 1. Comparison between content, output, and content to output ratio between controls for written samples and controls for spoken samples

	Measures	Control written	Control spoken
**Group *n***		20	20
**CU**	Average±SD	18.5±9.32	29.35±6.03
	Minimum - maximum	6 - 32	19 - 41
	95% CI*	-14.94 to -6.76	
	*p***	<0.001	
**UNITS**	Average±SD	61.7±27.86	136.85±46.59
	Minimum - maximum	21 - 98	67 - 236
	95% CI*	-106.27 to -44.03	
	*P***	<0.001	
**CU/U RATIO**	Average±SD	0.30±0.05	0.23±0.05
	Minimum - maximum	0.18 – 0.35	0.15 – 0.29
	95% CI*	-0.00083 to 0.14	
	*P***	0.052	

CU = Content Units; CU/U Ratio = Content Unit/Unit Ratio; SD=Standard deviation
*CI = Tukey's HSD 95% confidence interval: comparing the written vs spoken modality between written and spoken control groups
**Between ANOVA, Tukey's post-hoc test between both control samples though also considering PPA groups data

Supplementary Figure 1. Graphic comparison between written and spoken samples in control participants (written and spoken) and PPA.

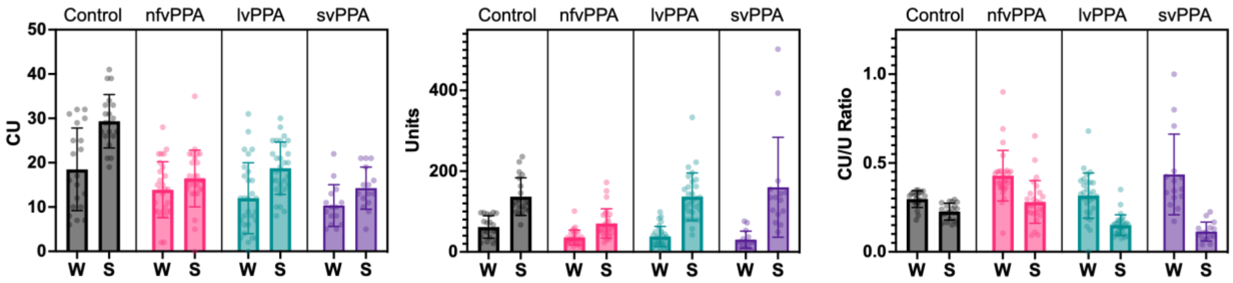

W=written; S=spoken, CU=Content Units, CU/U Ratio=Content Unit/Unit Ratio

Supplementary Table 2. Shapiro-Wilks test for normality distribution per group and type of measurement. Highlighted in red are those groups/measurements without a normal distribution.

Language Modality	Written		Spoken	
Measure	CU	Units	CU	Units
Groups				
Control Spoken			W(20) = 0.966 p = 0.6632	W(20) = 0.902 p = 0.0445
Control Written	W(20) = 0.904 p = 0.0501	W(20) = 0.900 p = 0.0413		
nfvPPA	W(28) = 0.968 p = 0.5374	W(28) = 0.877 p = 0.0035	W(28) = 0.916 p = 0.0275	W(27) = 0.878 p = 0.0044
lvPPA	W(30) = 0.953 p = 0.2066	W(30) = 0.921 p = 0.0289	W(30) = 0.974 p = 0.6546	W(30) = 0.938 p = 0.0805
svPPA	W(17) = 0.887 p = 0.0419	W(17) = 0.833 p = 0.0059	W(17) = 0.949 p = 0.4370	W(17) = 0.725 p = 0.0002
